# Senile Osteoarthritis Regulated by the Gut Microbiota: From Mechanisms to Treatments

**DOI:** 10.3390/ijms26041505

**Published:** 2025-02-11

**Authors:** Fan Yu, Chenyu Zhu, Wei Wu

**Affiliations:** 1 School of Exercise and Health, Shanghai University of Sports, Shanghai 200438, China; 13734934551@163.com (F.Y.); zhuchenyu156@163.com (C.Z.); 2 School of Athletic Performance, Shanghai University of Sports, Shanghai 200438, China

**Keywords:** gut microbiota, aging, osteoarthritis, therapeutic strategies

## Abstract

Osteoarthritis (OA) is a chronic, progressive degenerative joint disease that affects the entire synovial joint, leading to the progressive degeneration of articular cartilage. It seriously affects the quality of life and global disability of patients. OA is affected by a variety of factors; the most significant risk factor for OA is age. As individuals age, the risk and severity of OA increase due to the exacerbation of cartilage degeneration and wear and tear. In recent years, research has indicated that the gut microbiota may play a significant role in the aging and OA processes. It is anticipated that regulating the gut microbiota may offer novel approaches to the treatment of OA. The objective of this paper is to examine the relationship between the gut microbiota and senile OA, to investigate the potential mechanisms involved. This review also summarizes the therapeutic strategies related to gut flora in OA management, such as prebiotics and probiotics, diet, exercise, traditional Chinese medicine (TCM) modification, and fecal microbiota transplantation (FMT), highlighting the potential clinical value of gut flora and elucidating the current challenges. The foundation for future research directions is established through the summarization of current research progress.

## 1. Introduction

Osteoarthritis (OA) is a common chronic degenerative disease characterized by degenerative changes in cartilage, destruction of subchondral bone, and bone redundancy [1]. The most typical clinical manifestations of the disease are joint deformity, slowly progressive joint pain, and limited mobility, which can impose a serious burden on patients’ quality of life and socioeconomics [2]. OA is the fourth leading cause of disability worldwide [3]. It is estimated that by 2050, nearly 1 billion people will suffer from OA [4]. The pathogenesis of OA has not yet been clarified, and gender, aging, obesity, injury, and overuse may be susceptible factors for OA [5,6]. For instance, women have a higher probability of developing OA compared to men [7]. Moreover, female OA patients frequently experience more severe joint pain and increased disability compared to male OA patients [8]. Obesity and overweight have long been recognized as potential risk factors for OA, particularly knee osteoarthritis (KOA) [9]. Evidence suggests that for every 5 kg increase in body weight above normal, the risk of knee OA increases by 36% [10]. Furthermore, in the younger demographics, injuries such as ligament strains and meniscus injuries are the primary cause of knee OA, increasing the risk of developing OA by more than sixfold [11]. Aging is widely acknowledged as the foremost risk factor for OA [12]. With the increase in global aging, OA is gradually developing into a major public health problem and becoming one of the main causes of pain and disability in the elderly [12]. According to the progress of clinical research and epidemiology, the link between aging and OA has been gradually confirmed [13].

The gut microbiota, located in the host’s intestine, is a collection of intestinal microbial populations consisting of bacteria, viruses, fungi, parasites, archaea, and protozoa [14], which play an important role in maintaining nutrient absorption, metabolic homeostasis, resistance to infections and pathogen invasions, and promotion of the development and maturation of the immune system [15,16]. However, these physiological functions may be impaired by changes in microbial composition, termed ‘gut microbiota dysbiosis’. Gut microbiota dysbiosis refers to the disruption of the intestinal microecological balance in response to a range of external factors (e.g., diet, infections, age, and genetics), resulting in alterations in the structure, function, and diversity of the intestinal microbiota, leading to a variety of pathological conditions and diseases [17,18]. Increasing evidence suggests a strong association between the gut microbiota and some factors associated with OA, such as aging, obesity, diet, and gender [19,20]. Aging has been shown to increase the incidence of OA [21]. Whereas the gut microbiota changes continuously from infancy to old age during co-evolution with the host, organismal aging is inevitably accompanied by changes in the gut microbiota [22]. The intestines of centenarians are enriched with Aspergillus phylum, in which are located many potentially pathogenic bacteria that can induce elevated levels of pro-inflammatory factors, leading to the development of inflammatory diseases such as OA [23].

Therefore, we review the key findings of the association between aging and OA and summarize the potential mechanisms by which gut microbes and their associated components influence aging OA. In addition, we summarize potential therapeutic approaches to prevent and treat OA by targeting gut microbes to aid in the amelioration of OA.

## 2. The Regulatory Role of Aging in OA

### 2.1. Pathological Mechanisms of OA

Traditionally, OA has been considered a total joint disease characterized by degeneration of articular cartilage. An increasing body of evidence indicates that, in addition to cartilage tissue, pathological changes in OA involve multiple tissue systems, including the synovium and subchondral bone.

#### 2.1.1. Articular Cartilage

Articular cartilage covers the articular surface of the bones in a joint and consists mainly of chondrocytes and extracellular matrix (ECM). It is a smooth, elastic, translucent connective tissue that withstands loads and reduces joint friction [24]. The ECM is the main component of cartilage tissue and consists mainly of high levels of water, collagen fibers, and proteoglycans, which can provide structural and biochemical support to chondrocytes [25]. The earliest pathological changes of OA are usually seen on the surface of the articular cartilage. The chondrocytes are the only type of cell in the articular cartilage, and they are highly susceptible to damage due to the lack of blood vessels, lymphatic fluids, and nerves. Articular cartilage takes a long time to heal after injury, which is an important factor in causing OA [26]. In the early stage, OA chondrocytes are mainly effector chondrocytes and regulatory chondrocytes, while in the late stage, they evolve into mast cells and are accompanied by an increase in collagen type II alpha 1 chain (COL2A1), matrix metalloproteinase-9 (MMP9), and MMP13, which leads to an increase in chondrocyte size and matrix-degrading enzymes, leading to remodeling and mineralization of cartilage [27,28]. MMP13 plays a central role in cartilage catabolism [29]. In OA chondrocytes, high levels of MMP13 expression degrade collagen types I, II, and III, resulting in accelerated chondrolysis and lesions [30,31]. Apoptosis is a common phenomenon in OA [32]. The number of apoptotic deaths increases with the severity of the lesion and is considered a late event in OA, closely associated with cartilage destruction and matrix degradation [33,34].

#### 2.1.2. Subchondral Bone

The subchondral bone consists of the subchondral bone plate and the subchondral trabeculae. The subchondral bone plate is located below the calcified cartilage layer and is a thin cortical plate with an obvious void structure through which blood vessels and nerves pass, which is an important channel connecting cartilage and subchondral bone [35,36]. The subchondral trabeculae are located below the subchondral bone plate and play an important role in cartilage nutrient supply and metabolism, joint shock absorption, and structural support [37]. The progression of cartilage degradation in OA is closely related to bone remodeling and sclerosis of the subchondral bone. In the early stages of OA, abnormal biomechanical and biochemical factors recruit and promote osteoclast differentiation, leading to increased subchondral bone porosity, decreased bone volume, decreased bone density, and bone remodeling [38]. This abnormal bone remodeling not only reduces the stability and stress tolerance of the joints but also causes joint pain in OA [39]; advanced stages of OA favor increased bone formation and decreased bone resorption. Excessive bone formation causes a significant increase in subchondral bone mineral density and bone volume, resulting in the formation of osteosclerosis [40]. Furthermore, bone remodeling and osteosclerosis will change the mechanical and mechanical properties of subchondral bone, causing its absorption shear force and cushioning force to weaken, directly or indirectly leading to cartilage degeneration and accelerating disease progression [41].

#### 2.1.3. Synovial Tissue

Although the pathogenesis of OA is still unclear, with the in-depth study of OA, more and more research data suggest that OA is a joint disease closely related to synovial tissue [42]. Synovium is a special connective tissue that maintains synovial fluid and nourishes chondrocytes mainly by producing lubricin and hyaluronic acid [43]. The synovium can be divided into an inner synovial layer and a sub-synovial layer, which contains synovial macrophages (SMs) and synovial fibroblasts (FLSs) [44]. Histologically, the synovium in patients with OA is characterized by synovial intimal hyperplasia and subintimal fibrosis with an inflammatory cell infiltrate consisting of macrophages and lymphocytes [45,46]. An increase in M1 pro-inflammatory cytokines including interleukin-1β (IL-1β) and tumor necrosis factor-alpha (TNF-α), and a decrease in M2 anti-inflammatory cytokines such as IL-10, induce osteoclastogenesis [47]. FLSs contribute to cartilage degradation by promoting the release of inflammatory or catabolic and anabolic factors [48]. Especially in adult OA patients, FLSs are more responsive to pro-inflammatory stimuli and can secrete a variety of protein hydrolases (MMP3, MMP13, etc.) to participate in cartilage degradation, and a variety of inflammatory mediators and chemokines can also induce FLSs to produce protein hydrolases, exacerbating cartilage destruction [49]. In addition to the production of synovial fluid and lubricin to maintain joint homeostasis, the synovial lymphatic system (SLS) is located within the synovial tissues and promotes the breakdown and metabolism of inflammatory factors. This, in turn, maintains intra-articular homeostasis by ensuring a dynamic balance of synovial fluid components under both physiological and pathological conditions [50].

### 2.2. The Role of Aging in the Regulation of OA

In recent years, many studies have reported that senescent cells gradually accumulate in aged cartilage and participate in the development of OA [51]. When senescent cells from mouse ear cartilage were injected into the knee joint cavity, it was observed that these cells caused leg pain and activity dysfunction and ultimately induced the development of KOA in mice [52]. This study demonstrated for the first time that cellular senescence can cause OA. There is a large accumulation of senescent cells in the articular cartilage and synovium of patients with anterior cruciate ligament transection (ACLT), and the selective removal of these cells can promote chondrogenesis, reduce the inflammatory factor IL-6 and expression of MMP3, and alleviate the progression of post-traumatic OA [53].

#### 2.2.1. Senescence-Associated Secretory Phenotypes

One of the mechanisms by which aging promotes chronic inflammation is through cellular senescence. Cellular senescence is one of the hallmarks of aging and has been shown to accumulate in various tissues with age [54]. Chondrocytes are the only cell type found in articular cartilage. Chondrocyte senescence has been identified as a key factor in causing OA and is characterized by cellular growth cycle arrest, resistance to apoptosis, and sustained secretion of senescence-associated secretory phenotypes (SASPs) [55,56]. The SASP is an umbrella term for pro-inflammatory cytokines, chemokines, and matrix metalloproteinases involved in the destruction of joint tissues and is activated in conjunction with the dynamic build-up of the aging process [57,58]. SASPs are activated through the promotion of the secretion of inflammatory mediators such as IL-1β, TNF-α, IL-6, and MMPs to exacerbate the inflammatory response and stimulate neighboring cellular senescence, thus leading to pathological changes in senescent OA [59,60]. MMP13 secreted by SASPs is mainly present in cartilage, and in addition to being able to degrade collagen, it also degrades the proteoglycan molecule in aggregated glycans, and thus has a dual matrix-damaging role and is a major player in the degenerative process in the pathogenesis of OA [61]. Vascular endothelial growth factor (VEGF) is one of the important members of SASPs and is an essential cytokine in the process of osteogenesis within cartilage [62]. VEGF acts as a promoter of osteoclastogenesis, inducing the up-regulation of the expression of MMP2 and MMP9, and promotes chondrocyte apoptosis [63]. VEGF and its cognate receptors are abundantly expressed in OA cartilage, which can lead to osteoclastogenesis and play an important role in the pathological process of OA [64,65]. It is noteworthy that vascular endothelial growth factor C (VEGF-C), a growth factor implicated in the proliferation, differentiation, and survival of lymphatic endothelial cells (LECs), has been shown to improve SLS function and attenuate age-related progression of OA [66]. In addition, IL-6 levels increase with age and are strongly associated with diseases of aging, and are important inflammatory mediators in OA [67].

#### 2.2.2. Aging and Oxidative Stress

Oxidative stress is a key factor contributing to OA. Oxidative stress is the result of an imbalance between the production of reactive oxygen species (ROS) and their removal through antioxidant defenses [68]. ROS are mainly derived from chondrocytes, where they are usually present at low levels and are important for maintaining cellular homeostasis and function [69]. With age, increased oxidative stress and inflammation associated with senescence promote the accumulation of senescent cells and reduce chondrocyte survival and response to growth factors, making oxidative stress a major cause of stress-induced senescence during aging [70,71]. Oxidative stress levels are significantly elevated in OA cartilage, which is a major cause of chronic inflammation and a consequence of ROS overproduction [72]. On the one hand, ROS up-regulates matrix metalloproteinases and induces the expression of inflammatory factors such as nitric oxide synthase (NOS), IL-6, IL-1, and TNF-α, which promote the inflammatory pathological manifestations of OA [73]; on the other hand, the high up-regulation of cytokines, such as IL-1β, TNF-α, and IL-6 in OA joints will induce ROS production and expression of matrix-degrading proteases, leading to ECM degradation and joint dysfunction [72]. NO is one of the major components of ROS [74]. It has been reported that NO increases inflammatory responses by stimulating the activation of the nuclear factor-κB (NF-κB) signaling pathway, which promotes the production of TNF-α and IL-1β [75]. In turn, inflammatory factors lead to increased ROS production in mitochondria and chondrocytes, which triggers SASPs through activation of p38 MAPK, further promoting chondrocyte senescence and chondrocyte apoptosis [76,77].

#### 2.2.3. Aging and Mitochondrial Damage

Mitochondria are the energy-generating centers within the cell and perform a variety of biochemical processes including regulation of metabolic activities and apoptosis [78]. Mitochondrial dysfunction refers to mitochondrial DNA damage, decreased mitochondrial membrane potential, decreased oxidative phosphorylation, increased ROS production, etc. These changes lead to abnormal mitochondrial function, affecting normal cellular function and metabolism, which is regarded as one of the ‘hallmarks of aging’ [79,80]. It has been found that mitochondrial dysfunction causes oxidative stress, leading to excessive accumulation of intracellular ROS and an imbalance in cellular antioxidant capacity [81]. Excessive ROS can damage cell membranes, disrupting the integrity of cellular structures and damaging lysosomal membranes, causing lysosomal release and hydrolysis of intracellular substances, resulting in chondrocyte death [82,83]. In addition, mitochondrial oxidative stress induces the release of MMPs and degradation of the ECM [84], which is important for maintaining the normal structure and function of chondrocytes, and its degradation leads to structural damage and loss of function of chondrocytes, accelerating the degenerative lesions of articular cartilage and the induction of OA [85]. Chronic inflammation is another hallmark of aging [86]. Mitochondrial damage activates Nod-like receptor thermal protein domain associated protein 3 (NLRP3) inflammatory vesicles, leading to excessive release of inflammatory mediators such as IL-1β and TNF-α, exacerbating the inflammatory response of chondrocytes and promoting chondrocyte apoptosis [87,88].

#### 2.2.4. Aging and Autophagy

Autophagy is an intracellular process of self-degradation, which in most cases is a protective mechanism that maintains cellular integrity by removing damaged macromolecules and organelles and plays an important role in cartilage growth and development and the maintenance of articular cartilage homeostasis [89,90]. Stimulation of autophagy has anti-aging effects, preventing chondrocyte death and cartilage senescence and promoting longevity [91,92]. However, with age, the basic autophagic activity of cells in senescent tissues decreases, and the clearance efficiency decreases, leading to increased aggregation of various macromolecular proteins [93], which attenuates the protective effect on cartilage and ultimately results in cartilage degeneration and a series of aging-related diseases [94]. In advanced stages of OA, chondrocytes exhibit decreased autophagy and increased apoptosis [95]. Autophagy-related proteins, such as unc-51-like kinase 1 (ULK1), Beclin-1, and light chain 3 (LC3), are highly expressed in clusters of human chondrocytes, whereas their expression is reduced in the elderly [96]. Decreased autophagy leads to reduced chondrocyte activity and accelerated chondrocyte death, which may be the underlying cause of OA development [97]. In the early stage of OA, the enhancement of autophagic activity in superficial chondrocytes may serve as an adaptive response to avoid cell death. In contrast, the absence of autophagy in deep chondrocytes may be associated with abnormal calcification of cartilage and replacement of chondrocytes in late OA [98]. In addition, decreased autophagy in senescent cells promotes oxidative stress and ROS production [99]. Accumulation of ROS accelerates the onset of age-related degenerative diseases [100].

#### 2.2.5. Aging and the ECM

**Figure 1:**
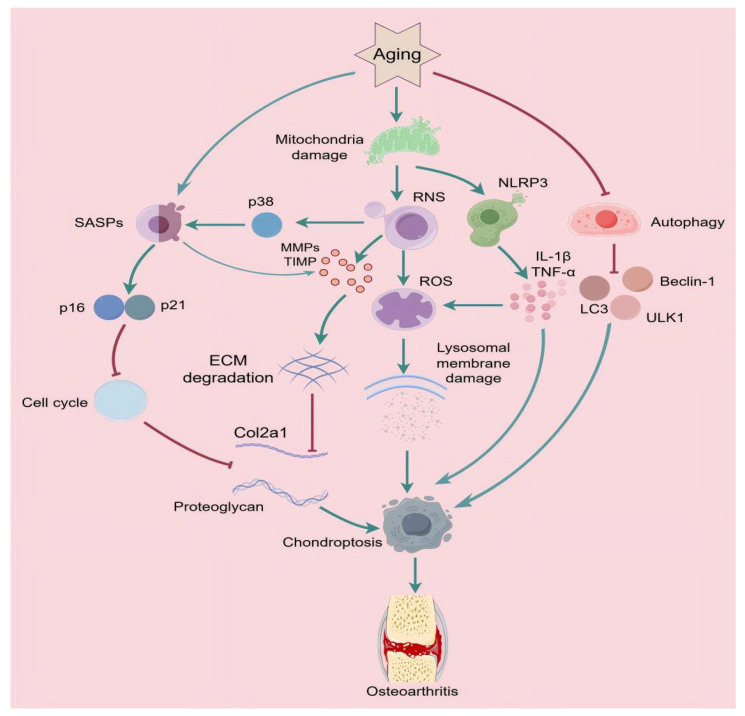
The mechanism of osteoarthritis in aging. The process of senescence results in the induction of apoptosis through the activation of SASPs, mitochondrial damage, and impaired autophagy. This leads to an increase in the production of ROS, inflammatory factors (IL-1β and TNF-α), and MMPs, as well as the promotion of ECM degradation and the release of hydrolytic enzymes. SASPs inhibit the cell cycle through the promotion of p16 and p21, and the inhibition of autophagy by senescence results in decreased expression of autophagy-related proteins (LC3, Beclin-1, ULK1). This ultimately promotes apoptosis and leads to OA.

The ECM is a complex meshwork of various proteins, including collagen, fibronectin, and proteoglycans, that regulates cellular homeostasis, confers biomechanical properties to articular cartilage, and provides mechanical support [101]. As cells age, the ECM will undergo alterations including transcriptional, translational, and post-translational modifications of its components, which directly or indirectly affect the interaction between the ECM and the cell, resulting in corresponding damage [102]. For example, the integrity of the ECM is decreased by glycosylation of collagen fibers, oxidation, inappropriate cross-linking, and accumulation of protein macromolecules [103]. Meanwhile, the accumulation of senescent cells directly affects ECM synthesis, secretion, and remodeling [104]. With cellular senescence, the up-regulation of two deterrent proteins, p16, and p21, will irreversibly stall the cell cycle in the G0 or G1 phase, preventing the cell from replicating and affecting the transcription and translation of ECM genes, leading to alterations in the composition and structure of the ECM [105]. ECM-degrading enzymes are up-regulated in senescent cells. Senescent cells affect the structure and content of the ECM by secreting the corresponding SASPs, in particular through the up-regulation of MMPs and tissue inhibitor of metalloproteinases (TIMP), leading to gradual degradation and dysfunction of the ECM and accelerated joint destruction [106,107] (Figure 1).

## 3. Gut Microbiota Regulates Senile OA

It is widely accepted that dysregulation of the gut microbiota represents a significant contributing factor to the development of OA [108]. In recent years, an increasing number of studies have demonstrated a strong correlation between gut microbiota and osteoarthritis. It has been shown that alterations in the gut microbiota can transmit signals through the ‘gut–joint axis’, which directly or indirectly influences the inflammatory response of the joints and disease progression by modulating the intestinal mucosal barrier, intestinal metabolites, and the level of immunity and disease progression [109].

### 3.1. Intestinal Mucosal Barrier

The composition of the gut microbiota plays an essential role in regulating the function of the intestinal mucosal barrier [110]. The intestinal mucosal barrier serves to prevent the penetration of noxious substances from the intestinal lumen into the blood circulation, thereby maintaining the overall health of the organism [111]. The process of aging not only affects the composition of the intestinal flora but is also associated with changes in the intestinal mucosa and barrier function. These changes include a contraction of the protective intestinal mucus layer, loss of intestinal tight junction proteins, and increased permeability of the intestinal epithelial barrier [112]. This leads to the leakage of toxic bacterial metabolites into the circulation, which in turn promotes the development of low-grade inflammation throughout the body [113]. Thevaranjan et al. [114] transplanted the microbial microflora of an aged mice population into young mice, which resulted in increased intestinal permeability in the young mice. This suggests that the composition of the microbiota can influence intestinal permeability and that permeability increases with age. The tight junctions between the intestinal epithelial cells are permitted to disintegrate, resulting in augmented absorption of bacterial products, including lipopolysaccharides [115], and the stimulation of inflammatory responses in vivo [116]. It has been demonstrated that elevated levels of LPS in the blood can activate janus tyrosine kinase 2/signal transducer and activator of transcription 1 (JAK2/STAT1) by inducing the toll-like receptor 4 (TLR4) pathway, which in turn promotes macrophage M1 polarization [117,118]. This process leads to the release of pro-inflammatory cytokines, including IL-1, IL-6, IL-12, TNF-α, ROS, and NO, which exacerbate tissue inflammation and damage [119]. Alternatively, LPS has been demonstrated to induce the secretion of degradative enzymes, including MMPs, a disintegrin, and metalloproteinase with thrombospondin motifs 4 (ADAMTS4) and ADAMTS5 through the activation of NF-κB transactivation, which ultimately leads to the degradation of articular cartilage [120,121]. Furthermore, it has been demonstrated that LPS stimulates the synthesis of inflammatory and catabolic factors by increasing the expression of cyclooxygenase-2 (COX-2), prostaglandin E2 (PGE2), and NO, which ultimately results in chondrocyte apoptosis [121]. Therefore, the aforementioned studies indicate that elevated intestinal permeability resulting from disruption of the intestinal mucosal barrier can lead to the release of a considerable number of inflammatory factors, which ultimately exacerbates joint inflammation.

### 3.2. Immune Response

With age, senescent cells down-regulate proliferation-related genes while overexpressing inflammatory factors and other molecules that regulate the immune response thereby causing dysregulation of the immune system, leading to diseases associated with immune decline and chronic inflammation [122,123]. The gut microbiota plays a crucial role in the formation of the intestinal immune system and can modulate the immune response through the production of substances with immunomodulatory and anti-inflammatory functions such as short-chain fatty acids, indoles and their derivatives, and secondary bile acids (BAs) [124]. A disruption in the equilibrium between the gut microbiota and the immune system can result in damage to the intestinal immune system due to altered intestinal mucosal permeability. This, in turn, can lead to a dysregulation of the intestinal endo-environment and a variety of immune-mediated and related diseases (e.g., inflammatory bowel disease, rheumatoid arthritis, and OA) [125,126]. The levels of LPS, a metabolite derived from the gut microbiota, have been demonstrated to correlate with the severity of OA [127]. LPS can act on macrophages and neutrophils in the immune system through the activation of the CD14-LPS-LBP complex and the binding of TLR4, which leads to the conversion of macrophages to M1-type macrophages, inducing the synthesis of pro-inflammatory factors, such as IL-1β, TNF-α, and MMPs, which can initiate a pro-inflammatory response in immune cells, leading to significant secondary effects in the joint tissues, exacerbating the onset and progression of OA [128,129,130]. TLRs are present in a variety of innate immune cells and represent a significant class of protein molecules involved in the innate immune response [131]. They facilitate the interconnection between the intestinal barrier, intestinal flora, and the innate immune system, thereby influencing the development of the gut microbiota [132]. TLR4 induces an inflammatory response in chondrocytes by increasing the expression of inflammatory factors, such as IL-1β and MMPs, and decreasing the synthesis of proteoglycans and col2a1. This results in the degradation of the cartilage matrix and an exacerbation of OA severity [133,134]. Bifidobacteria have been demonstrated to promote immune responses and to produce metabolites such as indoleacetic acid (IAA) and indoleacrylic acid (IA). These metabolites have been shown to promote the integrity of the intestinal mucosal barrier and to inhibit inflammatory responses by stimulating IL-22 expression and activating the aryl hydrocarbon receptor (AhR) [135,136]. Conversely, the number of bifidobacteria is significantly reduced in the elderly, which induces an increase in inflammatory factors and produces inflammatory lesions such as OA [137]. *Therefore, the process of aging results in disturbances within the intestinal microecological environment, which can subsequently lead to the dysregulation of the immune response and the subsequent development of OA.*

### 3.3. Metabolites of the Gut Microbiota

**Figure 2:**
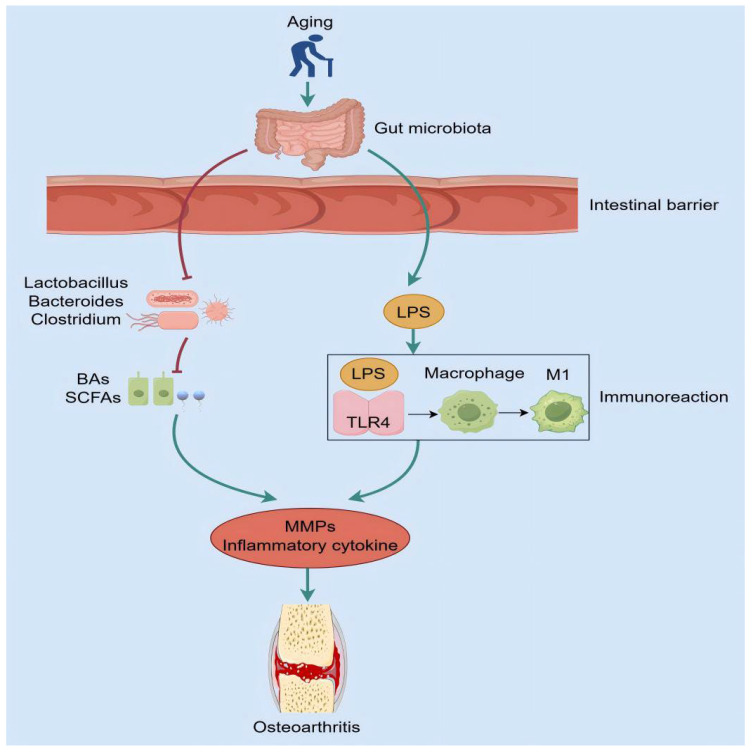
The mechanism of aging on gut microbiota. Aging will increase intestinal permeability and cause intestinal flora disorder. On the one hand, it will reduce the number of beneficial microbiota (Lactobacillus, Bacteroides, Clostridium), reduce the level of BAs and SCFAs, and promote the expression of inflammatory factors; on the other hand, the combination of LPS and TLR4 acts on macrophages, leading to the transformation of macrophages into M1 macrophages, triggering inflammatory reactions in immune cells, and jointly inducing the occurrence of OA.

The gut microbiota is capable of producing a diverse range of metabolites, and the levels of specific metabolites have been associated with the severity of inflammatory processes in arthritis [124]. BAs are metabolites derived from gut flora including species such as Lactobacillus, Bifidobacterium, Clostridium, and Anaplasma. They represent the most significant class of gut flora metabolites [138]. *BAs represent a principal pathway for cholesterol and lipid metabolism and have been linked to a range of metabolic disorders* [139]. It has been demonstrated that the gut microbiota in aged mice is markedly disrupted, exhibiting dysregulated BA homeostasis and markedly reduced levels of taurocholic acid (TCA) and taurohyodeoxycholic acid (THDCA). This results in a reduction in the abundance of BAs in mice, leading to increased systemic inflammation and a worsening of the progression of arthritis [140]. Furthermore, NLRP3 is an inflammatory vesicle that triggers an inflammatory response by activating cysteine-aspartate specific proteinase-1 (Caspase-1), thereby inducing the release of pro-inflammatory factors IL-1β and IL-18 [141]. It has been demonstrated that BAs inhibit the activation of NLRP3 inflammatory vesicles, which are responsible for inducing an inflammatory response [142]. Conversely, an increase in age has been shown to result in a reduction in BA levels, which in turn leads to an increase in the production of inflammatory cytokines [143]. These findings collectively indicate that a reduction in BA content associated with the aging process may contribute to the exacerbation of OA. It can therefore be surmised that age-related metabolic disorders of the gut microbiota may induce an inflammatory response, thereby exacerbating the progression of OA (Figure 2).

## 4. Potential Strategies for Targeting Gut Microbiota to Treat Senile OA

### 4.1. Diet

It is well established that diet plays a pivotal role in shaping the gut microbiota [144]. The supplementation of appropriate dietary fiber has been demonstrated to modulate the structure of the intestinal flora, thereby altering the composition of the microbiota, metabolism, and host immune response [123,145]. Resveratrol is a natural phenolic compound found in foods such as grapes, blueberries, and peanuts. It has been demonstrated to possess anti-inflammatory, antioxidant, and immunomodulatory effects [146,147]. It has been demonstrated that resveratrol markedly elevates the population of Bacteroides, Lactobacillus, and Bifidobacteria [148]. Similarly, Lactobacillus and Lactobacillus acidophilus (LAC) have been shown to enhance the bioavailability of resveratrol [149]. Increased resveratrol has been demonstrated to stimulate the regeneration of microvilli in the gut by modulating the nuclear factor erythroid 2-related factor 2 (Nrf2) and NF-κB signaling pathways, attenuating the increase in intestinal permeability induced by LPS and promoting tight junctions between the intestinal mucosal barriers [150]. Furthermore, resveratrol has been demonstrated to promote antioxidant stress by up-regulating the phosphatidylinositol 3-kinase (PI3K)/protein kinase B (AKT)-mediated Nrf2 signaling pathway, which in turn reduces intracellular ROS levels and apoptosis rates [151]. Additionally, resveratrol has been shown to inhibit the expression of pro-inflammatory cytokines such as IL-1β and TNF-α and enhances mitochondrial autophagy to reduce chondrocyte apoptosis and degradation, thereby exerting a protective effect against OA [152,153]. For instance, in the experiment of Gu et al. [154], the oral administration of resveratrol exerted anti-OA effects by reducing body weight, restoring the expression of type II collagen (COL2) in cartilage, and inhibiting chondrocyte apoptosis in mice fed a high-fat diet (HFD), which may be related to changes in the gut microbiota. The inhibitory effect on the number of cells undergoing apoptosis was more significant in the middle dose (22.5 mg/kg) and high dose resveratrol (45 mg/kg). Chondroitin sulfate (CS) is a nutraceutical that is widely used to improve the symptoms of OA and plays an important role in maintaining the structural integrity of cartilage tissue [155]. The oral administration of CS has been demonstrated to be associated with an increase in SCFAs and the Anaplasma phylum, and a decrease in the Aspergillus phylum [156,157]. Among these, the anaplastic gates were observed to significantly reduce the activity of pro-inflammatory cytokines in synovial fluid [158]. Furthermore, the activation of AMPK by SCFAs was demonstrated to induce inflammatory resistance, which could potentially attenuate the destruction of cartilage by inflammatory mediators and ameliorate joint damage [159]. Angelica dahurica, a botanical ingredient extracted from pine bark and metabolized by the gut microbiota, has been shown to reduce C-reactive protein levels by 71.3% and plasma free radical levels by 29.9% in patients with OA, with anti-inflammatory effects [160]. In a clinical trial, patients with primary OA were administered 150 mg of Angelica dahurica daily for a period of 60 days. The results demonstrated a significant reduction in pain, stiffness, physical functioning, and WOMAC scores by 18.6%, 18%, 19.6%, and 19.2%, respectively. This finding signifies a substantial alleviation of the clinical manifestations associated with OA [161]. In conclusion, diet may protect against OA by regulating the gut microbiota.

### 4.2. Probiotics and Prebiotics

Probiotics and prebiotics are dietary substances that have been demonstrated to improve host health by modulating the composition and function of the host’s gut microbiota, either directly or indirectly, by promoting the growth of beneficial flora [28,162]. Lactobacillus spp. have a long history of use as a probiotic therapy for OA [163]. Lactobacillus casei Shirota (LcS) is one of the most frequently utilized probiotics [164]. The ingestion of LcS has been demonstrated to reduce circulating levels of serum high-sensitivity C-reactive protein and to improve knee pain in patients with KOA [165]. The increased frequency of intake of LcS-containing fermented dairy products has been demonstrated to result in the attenuation of Helicobacter pylori-induced NF-κB activation, a reduction in pro-inflammatory cytokine levels (IL-6 and TNF-α), and an increase in fecal deoxycholic acid (CDCA), deoxycholic acid (DCA), litho-carbonic acid, and BAs abundance. This has been accompanied by a suppression of the inflammatory response [164,166]. *LAC, a probiotic commonly used in research*, has been demonstrated to reduce the relative abundance of Streptococcus species in the gut microbiome and decrease the amount of endotoxin produced by these bacteria [167]. This has been shown to alleviate cartilage degeneration in OA by rebalancing anti-inflammatory and pro-inflammatory factors, thereby reversing gut microecological dysregulation in OA models and pain [168]. Prebiotics are defined as foods that are rich in bioactive substances, including polysaccharides, oligofructose, and polyphenols [169]. Several animal studies have demonstrated that prebiotics can improve the status of osteoarthritis by modulating the gut microbiota [170,171]. For instance, the administration of continuous prebiotic oligofructose supplementation (10% *w*/*w*) over a period of two weeks has been shown to enhance the proliferation of beneficial bacterial species, including Actinobacteria and Bifidobacteria. Concurrently, this intervention has been observed to reverse the adverse effects of high-fat diet-induced obesity on the intestinal microbiota. Furthermore, it has been demonstrated to induce a reduction in pro-inflammatory mediators, such as IL-12 and monocyte chemotactic protein-1 (MCP-1), and to attenuate OARSI scores, thereby promoting the recovery of knee joint injury in mice models of OA [172]. Furthermore, Bifidobacterium longum CBi0703 lyophilized inactivated culture (LIC) administered at a dose of 1 μg/kg once a day for 12 weeks reduced cartilage structural damage and significantly lowered serum Coll2-1 levels, suggesting inhibition of COL2 degradation and a potentially preventive effect on the development of OA [170]. In conclusion, prebiotics or probiotics may prove effective in ameliorating the pathological process of OA by increasing the abundance of beneficial bifidobacteria.

### 4.3. Exercise

It has been demonstrated that exercise is beneficial to health and can effectively improve pain and function in patients with OA [173]. Furthermore, evidence indicates that exercise can markedly alter the structure, diversity, and abundance of intestinal flora, augment the number of beneficial microbial species, fortify intestinal mucosal immunity, and exert a beneficial influence on the intestinal microecological balance, energy homeostasis, and regulation [174,175]. Butyrate has been demonstrated to possess the capacity to attenuate cartilage degradation in vivo [176]. The increase in butyrate-producing bacteria, such as Faecalibacterium prausnitzii and Roseburia hominis, resulting from exercise leads to the promotion of chondrocyte autophagy through a reduction in necrotic apoptotic factors. Furthermore, the enhancement of LC3 and Beclin-1 expression, along with the up-regulation of the p62 protein, is also observed. Consequently, IL-1β-induced inflammatory cytokine expression and ECM degradation are attenuated, while ROS generation is effectively reduced, chondrocyte cell cycle arrest is prevented, and cartilage degradation is improved [177,178]. Furthermore, exercise has been demonstrated to confer benefits to OA joints by inhibiting the elevation of serum TNF-α, LPS, and endotoxin concentrations, and by inducing a decrease in systemic low-grade inflammation through a reduction in the abundance of Clostridium difficile [179]. Furthermore, the prolongation of exercise duration has been observed to elicit more pronounced alterations in the intestinal flora, which may potentially facilitate enhanced metabolic processes and mitigate the proliferation of flora, leaky gut, and systemic inflammation [180]. Petriz et al. [181] found that a training program involving running at a speed of 12.5 m per minute, five days per week for four weeks resulted in a 1.1-fold enhancement in the abundance of the Phylum Firmicutes, and a 1.8-fold reduction in the abundance of the Proteobacteria in obese rats. This finding is potentially valuable in the treatment of OA. In conclusion, exercise can prevent and delay the onset and progression of OA by regulating the metabolism of the intestinal microbiota and reducing the production of inflammatory factors.

### 4.4. Traditional Chinese Medicine

It is hypothesized that Chinese medicine may exert its therapeutic effects on osteoarthritic diseases by influencing and regulating the composition and balance of intestinal flora. One active ingredient of traditional Chinese medicine (TCM), quercetin, has a regulatory effect on the intestinal flora of OA rats. By increasing the levels of Lactobacillus spp. and Clostridium difficile, it can reduce the expression of TNF-α, IL-6, and MMP3 in the synovial membrane of OA. Furthermore, it can promote the expression of IL-10 and TIMP-3, enhancing anti-inflammatory responses in OA and promoting the protective effect on chondrocytes [163,182,183]. Erxian Decoction (EXD), a multi-component and multi-target herbal compound, has been demonstrated to improve articular cartilage degeneration by reducing the expression levels of MMP3 and MMP13 in KOA cartilage through the up-regulation of the abundance of Mycobacterium anisopliae and Mycobacterium thickeniens [184,185]. Moxibustion is a frequently employed treatment in the field of Chinese medicine, and has been demonstrated to alleviate systemic inflammation in patients with KOA by increasing the abundance of beneficial flora, including probiotics such as Lactobacillus and Blautia [186]. Furthermore, prolonged moxibustion treatment has been demonstrated to effectively regulate the dysfunction of intestinal flora, reducing the abundance of Rumen coccus and Parabacteroides spp. (which correlate with intestinal barrier disruption and OA severity) and down-regulating the levels of cartilage matrix-degrading enzymes (ADAMTS5 and MMP13) on the one hand to ameliorate cartilage degeneration and degradation, and at the same time, to promote a reduction in pro-inflammatory substances in the synovial fluid of KOA rats (IL-1β, TNF-α) to alleviate the progression of inflammation [187,188]. TNF-α and IL-1β are archetypal pro-inflammatory factors in KOA, and their activity and production can be inhibited by IL-10 [189,190]. In the study by Jia et al. [187], KOA mice were treated with moxibustion at two acupoints, “Dubi” and “Zusanli”, once every two days for a duration of 30 min. It was found that compared with the control group (no moxibustion), moxibustion for 4 weeks (MS4) and 6 weeks (MS6) not only increased the diversity and abundance of the intestinal flora in KOA mice, but also significantly contributed to the elevation of IL-10 levels (control: 9.93 ± 0.96 pg/mL vs. MS4: 15,96 ± 2,44 pg/mL vs. MS6: 13.72 ± 3.04 pg/mL), thus decreasing IL-1β and TNF-α levels, increasing the number of chondrocytes and improving cartilage surface roughness. In conclusion, TCM treats osteoarticular diseases and reduces inflammation by regulating the balance and composition of the gut microbiota.

### 4.5. Fecal Microbiota Transplantation

Fecal microbiota transplantation (FMT) represents an alternative therapeutic approach that is designed to treat diseases that are associated with the gut microbiota. This is achieved by transferring feces from a healthy donor to the distal gastrointestinal tract of a recipient patient, to reestablish the balance of the microbiota. This process is supported by the evidence presented in the reference [191]. FMT has demonstrated good therapeutic potential in a wide range of diseases, including autoimmune diseases, irritable bowel syndrome (IBS), inflammatory bowel disease (IBD), and metabolic diseases, and its efficacy has been widely validated in clinical practice [192,193,194,195]. For instance, a 20-year-old female patient suffering from rheumatoid arthritis (RA) who received a FMT from a healthy 8-year-old donor exhibited a gradual reduction in the required dosage of RA medications, along with a substantial improvement in her rheumatoid factor titer, disease activity, and disability index. Notably, no post-FMT discomfort was experienced by the patient [196]. As demonstrated by Huang et al. for the first time [197], it was shown that microbiomes from different populations could alter the pathological process of surgically induced OA in mice. In particular, the transplantation of fecal samples from OA patients with metabolic syndrome (Mets) into germ-free mice resulted in a significant elevation in low-grade inflammation, accompanied by an increase in the abundance of Fusobacterium and Faecalibacterium and a decrease in the abundance of Ruminococcaceae. This ultimately led to the exacerbation of the severity of surgically induced OA in meniscus ligament injuries in mice. This provides a promising avenue for the potential use of FMT in the treatment of OA. Regrettably, there is currently a paucity of studies in this field, and thus a need for extensive research to ascertain the viability of FMT as a treatment for OA (Table 1).

## 5. Conclusions and Perspectives

OA is a multifactorial disease, with aging representing the most significant risk factor. As individuals age, senescent cells gradually accumulate in articular cartilage, contributing to the pathological process of OA. The process of cellular senescence has been observed to promote the secretion of SASPs, increase ROS production and mitochondrial damage in chondrocytes, and induce the development of inflammatory responses and chondrocyte apoptosis. Autophagy plays a pivotal role in safeguarding chondrocytes against a multitude of stressors. Conversely, the process of aging has been observed to result in a reduction in autophagic activity, which in turn has been linked to an increase in oxidative stress generation and cartilage catabolism. Additionally, the ECM undergoes a progressive decline with age, resulting in impaired functionality and accelerated deterioration of articular cartilage.

Recent years have seen an increasing number of studies demonstrating the close relationship between a gut microbiota imbalance and the pathogenesis of OA. This review examines the relationship between intestinal flora and the progression of OA. It highlights that age-related changes in the intestinal flora disrupt the intestinal mucosal barrier and dysregulate the immune response, leading to the development of inflammatory lesions such as OA. Furthermore, a reduction in gut microbiota metabolites, such as BAs, has been observed to increase the levels of inflammatory cytokines, thereby exacerbating the progression of OA. A number of studies have indicated that modulation of the gut microbiota may represent a potential avenue for the treatment of OA. A range of therapeutic modalities, including diet, exercise, prebiotics and probiotics, TCM modification, and FMT, have been proposed as possible interventions to prevent and manage the progression of OA. In addition to acting alone, probiotics have been shown to combine with other substances to exert their therapeutic effects. So et al. [198] found that the combination of Lactobacillus casei (2 × 10^10^ cfu/kg, 500 mg/kg), glucosamine (250 mg/kg), and COL2 (250 mg/kg) enhanced anti-inflammatory effects in the joints and alleviated joint pain, which was effective in inhibiting the progression of OA. Furthermore, the combined effect of probiotic complex (12.5 mg/rat), zinc (20 mg/rat), and rosavin (100 mg/rat) reduced the expression of TNF-α, IL-6, and MMP3, while significantly increasing the levels of IL-10 and tissue inhibitor of metalloproteinase 3 (TIMP3). The inhibition of degenerative changes in cartilage and slowing down the progression of OA was also observed [199]. It is important to acknowledge that, despite its widespread recognition and acceptance as a treatment, FMT is not without its drawbacks. These include the risk of infection and a paucity of supporting data relating to long-term efficacy and safety [115]. Adverse events following FMT have been reported to be generally mild, self-limiting and gastrointestinal, with the majority manifesting as transient fever, nausea, abdominal discomfort, vomiting, diarrhea, and constipation [200]. However, it is important to note that FMT can also result in the development of serious adverse events, including death, sepsis, multi-organ failure, and recurrence of IBD [200]. The risk posed by FMT may be correlated with the donor’s intestinal flora [201]. For instance, a study revealed that at least one patient succumbed to multidrug-resistant *Escherichia coli* (*E. coli*) organisms following FMT treatment [202]. Consequently, there is a necessity for comprehensive and standardized screening of donors prior to FMT treatment, in addition to the management of potential complications, in order to mitigate the risk of FMT.

Given that the process of aging is ongoing, changes in the gut microbiota and its associated inflammatory state during aging may significantly influence the onset and development of OA as a key determinant. Nevertheless, the precise mechanism by which gut microbiota contributes to OA remains unclear. Further investigation is therefore required to substantiate the efficacy of regulating gut flora in the management of OA and to identify novel avenues for OA treatment. Furthermore, current studies on the gut microbiota in OA have focused on chondrocytes and lacked exploration of subchondral bone and synovial tissues. Consequently, future studies in this area should be strengthened to more comprehensively understand the role of gut microbiota in OA and to provide a scientific basis for the development of new therapies for OA.

## Figures and Tables

**Table 1:** Potential strategies for targeting the gut microbiota against OA.

Treatment	Gut Microbiota Regulation	Role	Ref.
Resveratrol	Bacteroides ↑ Lactobacillus ↑ Bacillus Bifidus ↑	Autophagy ↑ ROS ↓ Apoptosis ↓ Inflammation ↓	[148,151,152,153]
CS	Bacteroidetes ↑ SCFA ↑ Proteobacteria ↓	Inflammation ↓	[156,157,158]
LcS	BA ↑ Helicobacter pylori ↓	hs-CRP ↓ Inflammation ↓	[165,166]
LAC	Helicobacter pylori ↓ Streptococcus ↓	Endotoxin ↓ Inflammatory balance ↑	[168]
Oligofructose	Bifidobacteria ↑ Actinobacteria ↑	Inflammation ↓	[172]
Exercise	F.prausnitzii ↑ R.hominis ↑	Autophagy ↑ LC3 ↑ Beclin-1 ↑ Inflammation ↓ ECM degradation ↓	[177,178]
Exercise	Fusobacterium ↓	LPS ↓ Endotoxin ↓ Inflammation ↓	[179]
Quercetin	Lactobacillus genus ↑ Clostridium ↑	IL-10 ↑ TIMP-3 ↑ MMP3 ↓ Inflammation ↓	[163,182,183]
PAL	Bacteroidetes ↑ Firmicutes ↑	MMP3 ↓ MMP13 ↓	[184,185]
Moxibustion	Lactobacilli ↑ Blautia ↑	Mankin scores ↓ Inflammation ↓	[186]
Moxibustion	Ruminococcus ↓ Parabacteroides ↓	ADAMTS5 ↓ MMP13 ↓ Inflammation ↓	[187,188]
FMT	Fusobacterium ↑ Faecalibacterium ↑ Ruminococcaceae ↓	Endotoxin ↑ Low-grade inflammation ↑	[197]

ABBREVIATIONS: CS: Chondroitin sulfate, LCS: Lacticaseibacillus casei Shirota, LAC: Lactobacillus acidophilus, PAL: Palmatine, FMT: Fecal microbiota transplantation, hs-CRP: Hypersensitive C-reactive protein.

## Abbreviations

OA: Osteoarthritis, ECM: Extracellular matrix, COL2A1: Collagen type II alpha 1 chain, COL2: Type II collagen, MMP9: Matrix metalloproteinase-9, SM: Synovial macrophages, FLS: Synovial fibroblasts, IL-1β: Interleukin-1β, TNF-α: Tumor necrosis factor-alpha, KOA: Knee osteoarthritis, SLS: Synovial lymphatic system, ACLT: Anterior cruciate ligament transection, SASPs: Senescence-associated secretory phenotypes, VEGF: Vascular endothelial growth factor, VEGF-C: Vascular endothelial growth factor C, ROS: Reactive oxygen species, NOS: Nitric oxide synthase, NF-κB: Nuclear factor-κB, NLRP3: Nod-like receptor thermal protein domain associated protein 3, ULK1: Unc-51-like kinase 1, LC3: Light chain 3, TIMP: Tissue inhibitor of metalloproteinases, LPS: Lipopolysaccharides, JAK2/STAT1: Janus tyrosine kinase 2/signal transducer and activator of transcription 1, TLR4: Toll-like receptor 4, ADAMTS4: Thrombospondin motifs 4, COX-2: Cyclooxygenase-2, PGE2: Prostaglandin E2, BAs: Bile acids, IAA: Indoleacetic acid, IA: Indoleacrylic acid, Ahr: Aryl hydrocarbon receptor, TCA: Taurocholic acid, THDCA: Taurohyodeoxycholic acid, Caspase-1: Cysteine-aspartate specific proteinase-1, LAC: Lactobacillus acidophilus, Nrf2: Nuclear factor erythroid 2-related factor 2, PI3K: Phosphatidylinositol 3-kinase, AKT: Protein kinase B, CDCA: Deoxycholic acid, MCP-1: Monocyte chemotactic protein-1, LIC: Lyophilised inactivated culture, TCM: Traditional Chinese medicine, MS4: Moxibustion for 4 weeks, MS6: Moxibustion for 6 weeks, FMT: Fecal microbiota transplantation, IBS: Irritable bowel syndrome, IBD: Inflammatory bowel disease, RA: Rheumatoid arthritis, Mets: Metabolic syndrome, TIMP3: Tissue inhibitor of metalloproteinase 3, *E. coli*: *Escherichia coli*.

## References

[1] Fujii Y., Liu L., Yagasaki L., Inotsume M., Chiba T., Asahara H. (2022). Cartilage Homeostasis and Osteoarthritis. Int. J. Mol. Sci..

[2] Hunter D.J., Bierma-Zeinstra S. (2019). Osteoarthritis. Lancet.

[3] Yao Q., Wu X., Tao C., Gong W., Chen M., Qu M., Zhong Y., He T., Chen S., Xiao G. (2023). Osteoarthritis: Pathogenic signaling pathways and therapeutic targets. Signal Transduct. Target. Ther..

[4] Korzh O., GBD 2021 Osteoarthritis Collaborators (2023). Global, regional, and national burden of osteoarthritis, 1990–2020 and projections to 2050: A systematic analysis for the Global Burden of Disease Study 2021. Lancet. Rheumatol..

[5] Glyn-Jones S., Palmer A.J., Agricola R., Price A.J., Vincent T.L., Weinans H., Carr A.J. (2015). Osteoarthritis. Lancet.

[6] Lieberthal J., Sambamurthy N., Scanzello C.R. (2015). Inflammation in joint injury and post-traumatic osteoarthritis. Osteoarthr. Cartil..

[7] Peshkova M., Lychagin A., Lipina M., Di Matteo B., Anzillotti G., Ronzoni F., Kosheleva N., Shpichka A., Royuk V., Fomin V. (2022). Gender-Related Aspects in Osteoarthritis Development and Progression: A Review. Int. J. Mol. Sci..

[8] Laitner M.H., Erickson L.C., Ortman E. (2021). Understanding the Impact of Sex and Gender in Osteoarthritis: Assessing Research Gaps and Unmet Needs. J. Women’s Health.

[9] Felson D.T., Lawrence R.C., Dieppe P.A., Hirsch R., Helmick C.G., Jordan J.M., Kington R.S., Lane N.E., Nevitt M.C., Zhang Y. (2000). Osteoarthritis: New insights. Part 1: The disease and its risk factors. Ann. Intern. Med..

[10] Lementowski P.W., Zelicof S.B. (2008). Obesity and osteoarthritis. Am. J. Orthop..

[11] Snoeker B., Turkiewicz A., Magnusson K., Frobell R., Yu D., Peat G., Englund M. (2020). Risk of knee osteoarthritis after different types of knee injuries in young adults: A population-based cohort study. Br. J. Sports Med..

[12] Hawker G.A., King L.K. (2022). The Burden of Osteoarthritis in Older Adults. Clin. Geriatr. Med..

[13] Loeser R.F., Collins J.A., Diekman B.O. (2016). Ageing and the pathogenesis of osteoarthritis. Nat. Rev. Rheumatol..

[14] Rowland I., Gibson G., Heinken A., Scott K., Swann J., Thiele I., Tuohy K. (2018). Gut microbiota functions: Metabolism of nutrients and other food components. Eur. J. Nutr..

[15] Lynch J.B., Hsiao E.Y. (2019). Microbiomes as sources of emergent host phenotypes. Science.

[16] Al-Rashidi H.E. (2022). Gut microbiota and immunity relevance in eubiosis and dysbiosis. Saudi J. Biol. Sci..

[17] Petersen C., Round J.L. (2014). Defining dysbiosis and its influence on host immunity and disease. Cell. Microbiol..

[18] Chen Y., Zhou J., Wang L. (2021). Role and Mechanism of Gut Microbiota in Human Disease. Front. Cell. Infect. Microbiol..

[19] Biver E., Berenbaum F., Valdes A.M., Araujo de Carvalho I., Bindels L.B., Brandi M.L., Calder P.C., Castronovo V., Cavalier E., Cherubini A. (2019). Gut microbiota and osteoarthritis management: An expert consensus of the European society for clinical and economic aspects of osteoporosis, osteoarthritis and musculoskeletal diseases (ESCEO). Ageing Res. Rev..

[20] Geng J., Ni Q., Sun W., Li L., Feng X. (2022). The links between gut microbiota and obesity and obesity related diseases. Biomed. Pharmacother..

[21] Loeser R.F. (2017). The Role of Aging in the Development of Osteoarthritis. Trans. Am. Clin. Climatol. Assoc..

[22] Odamaki T., Kato K., Sugahara H., Hashikura N., Takahashi S., Xiao J.Z., Abe F., Osawa R. (2016). Age-related changes in gut microbiota composition from newborn to centenarian: A cross-sectional study. BMC Microbiol..

[23] Biagi E., Franceschi C., Rampelli S., Severgnini M., Ostan R., Turroni S., Consolandi C., Quercia S., Scurti M., Monti D. (2016). Gut Microbiota and Extreme Longevity. Curr. Biol. CB.

[24] Monteagudo S., Lories R.J. (2017). Cushioning the cartilage: A canonical Wnt restricting matter. Nat. Rev. Rheumatol..

[25] Michel G., Tonon T., Scornet D., Cock J.M., Kloareg B. (2010). The cell wall polysaccharide metabolism of the brown alga Ectocarpus siliculosus. Insights into the evolution of extracellular matrix polysaccharides in Eukaryotes. New Phytol..

[26] Hu W., Chen Y., Dou C., Dong S. (2021). Microenvironment in subchondral bone: Predominant regulator for the treatment of osteoarthritis. Ann. Rheum. Dis..

[27] Ji Q., Zheng Y., Zhang G., Hu Y., Fan X., Hou Y., Wen L., Li L., Xu Y., Wang Y. (2019). Single-cell RNA-seq analysis reveals the progression of human osteoarthritis. Ann. Rheum. Dis..

[28] Singh P., Marcu K.B., Goldring M.B., Otero M. (2019). Phenotypic instability of chondrocytes in osteoarthritis: On a path to hypertrophy. Ann. N. Y. Acad. Sci..

[29] Sprangers S., Everts V. (2019). Molecular pathways of cell-mediated degradation of fibrillar collagen. Matrix Biol. J. Int. Soc. Matrix Biol..

[30] Li X., Xie C., Xiao F., Su H., Li Z., Weng J., Huang Y., He P. (2022). Circular RNA circ_0000423 regulates cartilage ECM synthesis via circ_0000423/miRNA-27b-3p/MMP-13 axis in osteoarthritis. Aging.

[31] Kaneva M.K. (2022). Neutrophil elastase and its inhibitors-overlooked players in osteoarthritis. FEBS J..

[32] Blanco F.J., Guitian R., Vázquez-Martul E., de Toro F.J., Galdo F. (1998). Osteoarthritis chondrocytes die by apoptosis. A possible pathway for osteoarthritis pathology. Arthritis Rheum..

[33] Sharif M., Whitehouse A., Sharman P., Perry M., Adams M. (2004). Increased apoptosis in human osteoarthritic cartilage corresponds to reduced cell density and expression of caspase-3. Arthritis Rheum..

[34] Musumeci G., Loreto C., Carnazza M.L., Strehin I., Elisseeff J. (2011). OA cartilage derived chondrocytes encapsulated in poly(ethylene glycol) diacrylate (PEGDA) for the evaluation of cartilage restoration and apoptosis in an in vitro model. Histol. Histopathol..

[35] Burr D.B., Gallant M.A. (2012). Bone remodelling in osteoarthritis. Nat. Rev. Rheumatol..

[36] Castañeda S., Roman-Blas J.A., Largo R., Herrero-Beaumont G. (2012). Subchondral bone as a key target for osteoarthritis treatment. Biochem. Pharmacol..

[37] Holopainen J.T., Brama P.A., Halmesmäki E., Harjula T., Tuukkanen J., van Weeren P.R., Helminen H.J., Hyttinen M.M. (2008). Changes in subchondral bone mineral density and collagen matrix organization in growing horses. Bone.

[38] Findlay D.M., Atkins G.J. (2014). Osteoblast-chondrocyte interactions in osteoarthritis. Curr. Osteoporos. Rep..

[39] Zhu S., Zhu J., Zhen G., Hu Y., An S., Li Y., Zheng Q., Chen Z., Yang Y., Wan M. (2019). Subchondral bone osteoclasts induce sensory innervation and osteoarthritis pain. J. Clin. Investig..

[40] Azzini G.O.M., Santos G.S., Visoni S.B.C., Azzini V.O.M., Santos R.G.D., Huber S.C., Lana J.F. (2020). Metabolic syndrome and subchondral bone alterations: The rise of osteoarthritis—A review. J. Clin. Orthop. Trauma.

[41] Radin E.L., Rose R.M. (1986). Role of subchondral bone in the initiation and progression of cartilage damage. Clin. Orthop. Relat. Res..

[42] Sellam J., Berenbaum F. (2010). The role of synovitis in pathophysiology and clinical symptoms of osteoarthritis. Nat. Rev. Rheumatol..

[43] Scanzello C.R., Goldring S.R. (2012). The role of synovitis in osteoarthritis pathogenesis. Bone.

[44] Buckley C.D., Ospelt C., Gay S., Midwood K.S. (2021). Location, location, location: How the tissue microenvironment affects inflammation in RA. Nat. Rev. Rheumatol..

[45] Sanchez-Lopez E., Coras R., Torres A., Lane N.E., Guma M. (2022). Synovial inflammation in osteoarthritis progression. Nat. Rev. Rheumatol..

[46] Kuo S.J., Liu S.C., Huang Y.L., Tsai C.H., Fong Y.C., Hsu H.C., Tang C.H. (2019). TGF-β1 enhances FOXO3 expression in human synovial fibroblasts by inhibiting miR-92a through AMPK and p38 pathways. Aging.

[47] Chen Y., Jiang W., Yong H., He M., Yang Y., Deng Z., Li Y. (2020). Macrophages in osteoarthritis: Pathophysiology and therapeutics. Am. J. Transl. Res..

[48] Nair A., Kanda V., Bush-Joseph C., Verma N., Chubinskaya S., Mikecz K., Glant T.T., Malfait A.M., Crow M.K., Spear G.T. (2012). Synovial fluid from patients with early osteoarthritis modulates fibroblast-like synoviocyte responses to toll-like receptor 4 and toll-like receptor 2 ligands via soluble CD14. Arthritis Rheum..

[49] Chwastek J., Kędziora M., Borczyk M., Korostyński M., Starowicz K. (2022). Inflammation-Driven Secretion Potential Is Upregulated in Osteoarthritic Fibroblast-Like Synoviocytes. Int. J. Mol. Sci..

[50] Cao M., Ong M.T.Y., Yung P.S.H., Tuan R.S., Jiang Y. (2022). Role of synovial lymphatic function in osteoarthritis. Osteoarthr. Cartil..

[51] Coryell P.R., Diekman B.O., Loeser R.F. (2021). Mechanisms and therapeutic implications of cellular senescence in osteoarthritis. Nat. Rev. Rheumatol..

[52] Xu M., Bradley E.W., Weivoda M.M., Hwang S.M., Pirtskhalava T., Decklever T., Curran G.L., Ogrodnik M., Jurk D., Johnson K.O. (2017). Transplanted Senescent Cells Induce an Osteoarthritis-Like Condition in Mice. J. Gerontol. Ser. A.

[53] Jeon O.H., Kim C., Laberge R.M., Demaria M., Rathod S., Vasserot A.P., Chung J.W., Kim D.H., Poon Y., David N. (2017). Local clearance of senescent cells attenuates the development of post-traumatic osteoarthritis and creates a pro-regenerative environment. Nat. Med..

[54] Dodig S., Čepelak I., Pavić I. (2019). Hallmarks of senescence and aging. Biochem. Medica.

[55] McCulloch K., Litherland G.J., Rai T.S. (2017). Cellular senescence in osteoarthritis pathology. Aging Cell.

[56] Herranz N., Gil J. (2018). Mechanisms and functions of cellular senescence. J. Clin. Investig..

[57] Cuollo L., Antonangeli F., Santoni A., Soriani A. (2020). The Senescence-Associated Secretory Phenotype (SASP) in the Challenging Future of Cancer Therapy and Age-Related Diseases. Biology.

[58] Kuilman T., Michaloglou C., Vredeveld L.C., Douma S., van Doorn R., Desmet C.J., Aarden L.A., Mooi W.J., Peeper D.S. (2008). Oncogene-induced senescence relayed by an interleukin-dependent inflammatory network. Cell.

[59] Yue Z., Nie L., Zhao P., Ji N., Liao G., Wang Q. (2022). Senescence-associated secretory phenotype and its impact on oral immune homeostasis. Front. Immunol..

[60] Coppé J.P., Desprez P.Y., Krtolica A., Campisi J. (2010). The senescence-associated secretory phenotype: The dark side of tumor suppression. Annu. Rev. Pathol..

[61] Mehana E.E., Khafaga A.F., El-Blehi S.S. (2019). The role of matrix metalloproteinases in osteoarthritis pathogenesis: An updated review. Life Sci..

[62] Pfander D., Körtje D., Zimmermann R., Weseloh G., Kirsch T., Gesslein M., Cramer T., Swoboda B. (2001). Vascular endothelial growth factor in articular cartilage of healthy and osteoarthritic human knee joints. Ann. Rheum. Dis..

[63] Belotti D., Paganoni P., Manenti L., Garofalo A., Marchini S., Taraboletti G., Giavazzi R. (2003). Matrix metalloproteinases (MMP9 and MMP2) induce the release of vascular endothelial growth factor (VEGF) by ovarian carcinoma cells: Implications for ascites formation. Cancer Res..

[64] Hamilton J.L., Nagao M., Levine B.R., Chen D., Olsen B.R., Im H.J. (2016). Targeting VEGF and Its Receptors for the Treatment of Osteoarthritis and Associated Pain. J. Bone Miner. Res..

[65] Nagao M., Hamilton J.L., Kc R., Berendsen A.D., Duan X., Cheong C.W., Li X., Im H.J., Olsen B.R. (2017). Vascular Endothelial Growth Factor in Cartilage Development and Osteoarthritis. Sci. Rep..

[66] Lin X., Bell R.D., Catheline S.E., Takano T., McDavid A., Jonason J.H., Schwarz E.M., Xing L. (2023). Targeting Synovial Lymphatic Function as a Novel Therapeutic Intervention for Age-Related Osteoarthritis in Mice. Arthritis Rheumatol..

[67] Tyrrell D.J., Goldstein D.R. (2021). Ageing and atherosclerosis: Vascular intrinsic and extrinsic factors and potential role of IL-6. Nat. Rev. Cardiol..

[68] Pizzino G., Irrera N., Cucinotta M., Pallio G., Mannino F., Arcoraci V., Squadrito F., Altavilla D., Bitto A. (2017). Oxidative Stress: Harms and Benefits for Human Health. Oxidative Med. Cell. Longev..

[69] van Lent P.L., Nabbe K.C., Blom A.B., Sloetjes A., Holthuysen A.E., Kolls J., Van De Loo F.A., Holland S.M., Van Den Berg W.B. (2005). NADPH-oxidase-driven oxygen radical production determines chondrocyte death and partly regulates metalloproteinase-mediated cartilage matrix degradation during interferon-gamma-stimulated immune complex arthritis. Arthritis Res. Ther..

[70] Wu Z., Qu J., Zhang W., Liu G.H. (2024). Stress, epigenetics, and aging: Unraveling the intricate crosstalk. Mol. Cell.

[71] Ramasamy T.S., Yee Y.M., Khan I.M. (2021). Chondrocyte Aging: The Molecular Determinants and Therapeutic Opportunities. Front. Cell Dev. Biol..

[72] Ansari M.Y., Ahmad N., Haqqi T.M. (2020). Oxidative stress and inflammation in osteoarthritis pathogenesis: Role of polyphenols. Biomed. Pharmacother..

[73] Kim M.J., Woo S.W., Kim M.S., Park J.E., Hwang J.K. (2014). Anti-photoaging effect of aaptamine in UVB-irradiated human dermal fibroblasts and epidermal keratinocytes. J. Asian Nat. Prod. Res..

[74] Zorov D.B., Juhaszova M., Sollott S.J. (2014). Mitochondrial reactive oxygen species (ROS) and ROS-induced ROS release. Physiol. Rev..

[75] Ahmad N., Ansari M.Y., Haqqi T.M. (2020). Role of iNOS in osteoarthritis: Pathological and therapeutic aspects. J. Cell. Physiol..

[76] Bent E.H., Gilbert L.A., Hemann M.T. (2016). A senescence secretory switch mediated by PI3K/AKT/mTOR activation controls chemoprotective endothelial secretory responses. Genes Dev..

[77] Zhang B., Fu D., Xu Q., Cong X., Wu C., Zhong X., Ma Y., Lv Z., Chen F., Han L. (2018). The senescence-associated secretory phenotype is potentiated by feedforward regulatory mechanisms involving Zscan4 and TAK1. Nat. Commun..

[78] Duchen M.R. (2000). Mitochondria and calcium: From cell signalling to cell death. J. Physiol..

[79] Chen G., Kroemer G., Kepp O. (2020). Mitophagy: An Emerging Role in Aging and Age-Associated Diseases. Front. Cell Dev. Biol..

[80] Videla L.A., Marimán A., Ramos B., José Silva M., Del Campo A. (2022). Standpoints in mitochondrial dysfunction: Underlying mechanisms in search of therapeutic strategies. Mitochondrion.

[81] Kowalczyk P., Sulejczak D., Kleczkowska P., Bukowska-Ośko I., Kucia M., Popiel M., Wietrak E., Kramkowski K., Wrzosek K., Kaczyńska K. (2021). Mitochondrial Oxidative Stress-A Causative Factor and Therapeutic Target in Many Diseases. Int. J. Mol. Sci..

[82] Villalpando-Rodriguez G.E., Gibson S.B. (2021). Reactive Oxygen Species (ROS) Regulates Different Types of Cell Death by Acting as a Rheostat. Oxidative Med. Cell. Longev..

[83] Nagakannan P., Tabeshmehr P., Eftekharpour E. (2020). Oxidative damage of lysosomes in regulated cell death systems: Pathophysiology and pharmacologic interventions. Free Radic. Biol. Med..

[84] Liu H.M., Cheng M.Y., Xun M.H., Zhao Z.W., Zhang Y., Tang W., Cheng J., Ni J., Wang W. (2023). Possible Mechanisms of Oxidative Stress-Induced Skin Cellular Senescence, Inflammation, and Cancer and the Therapeutic Potential of Plant Polyphenols. Int. J. Mol. Sci..

[85] Peng Z., Sun H., Bunpetch V., Koh Y., Wen Y., Wu D., Ouyang H. (2021). The regulation of cartilage extracellular matrix homeostasis in joint cartilage degeneration and regeneration. Biomaterials.

[86] López-Otín C., Blasco M.A., Partridge L., Serrano M., Kroemer G. (2013). The hallmarks of aging. Cell.

[87] Zhang T., Ding S., Wang R. (2021). Research Progress of Mitochondrial Mechanism in NLRP3 Inflammasome Activation and Exercise Regulation of NLRP3 Inflammasome. Int. J. Mol. Sci..

[88] Li H., Yang X., Song Y., Zhu Q., Liao Z., Liang Y., Guo J., Wan B., Bao D. (2023). PRRSV infection activates NLRP3 inflammasome through inducing cytosolic mitochondrial DNA stress. Vet. Microbiol..

[89] Cao W., Li J., Yang K., Cao D. (2021). An overview of autophagy: Mechanism, regulation and research progress. Bull. Du Cancer.

[90] Takayama K., Kawakami Y., Kobayashi M., Greco N., Cummins J.H., Matsushita T., Kuroda R., Kurosaka M., Fu F.H., Huard J. (2014). Local intra-articular injection of rapamycin delays articular cartilage degeneration in a murine model of osteoarthritis. Arthritis Res. Ther..

[91] Hansen M., Rubinsztein D.C., Walker D.W. (2018). Autophagy as a promoter of longevity: Insights from model organisms. Nat. Rev. Mol. Cell Biol..

[92] Leidal A.M., Levine B., Debnath J. (2018). Autophagy and the cell biology of age-related disease. Nat. Cell Biol..

[93] Lim S.H.Y., Hansen M., Kumsta C. (2024). Molecular Mechanisms of Autophagy Decline during Aging. Cells.

[94] Lotz M.K., Caramés B. (2011). Autophagy and cartilage homeostasis mechanisms in joint health, aging and OA. Nat. Rev. Rheumatol..

[95] Lv X., Zhao T., Dai Y., Shi M., Huang X., Wei Y., Shen J., Zhang X., Xie Z., Wang Q. (2022). New insights into the interplay between autophagy and cartilage degeneration in osteoarthritis. Front. Cell Dev. Biol..

[96] Caramés B., Taniguchi N., Otsuki S., Blanco F.J., Lotz M. (2010). Autophagy is a protective mechanism in normal cartilage, and its aging-related loss is linked with cell death and osteoarthritis. Arthritis Rheum..

[97] Bouderlique T., Vuppalapati K.K., Newton P.T., Li L., Barenius B., Chagin A.S. (2016). Targeted deletion of Atg5 in chondrocytes promotes age-related osteoarthritis. Ann. Rheum. Dis..

[98] Almonte-Becerril M., Navarro-Garcia F., Gonzalez-Robles A., Vega-Lopez M.A., Lavalle C., Kouri J.B. (2010). Cell death of chondrocytes is a combination between apoptosis and autophagy during the pathogenesis of Osteoarthritis within an experimental model. Apoptosis.

[99] Mobasheri A., Matta C., Zákány R., Musumeci G. (2015). Chondrosenescence: Definition, hallmarks and potential role in the pathogenesis of osteoarthritis. Maturitas.

[100] Yang J., Luo J., Tian X., Zhao Y., Li Y., Wu X. (2024). Progress in Understanding Oxidative Stress, Aging, and Aging-Related Diseases. Antioxidants.

[101] Hynes R.O. (2009). The extracellular matrix: Not just pretty fibrils. Science.

[102] Xiao P., Zhang Y., Zeng Y., Yang D., Mo J., Zheng Z., Wang J., Zhang Y., Zhou Z., Zhong X. (2023). Impaired angiogenesis in ageing: The central role of the extracellular matrix. J. Transl. Med..

[103] Frantz C., Stewart K.M., Weaver V.M. (2010). The extracellular matrix at a glance. J. Cell Sci..

[104] Campisi J. (1998). The role of cellular senescence in skin aging. J. Investig. Dermatol. Symp. Proc..

[105] Varela-Eirín M., Demaria M. (2022). Cellular senescence. Curr. Biol. CB.

[106] Shin S.H., Lee Y.H., Rho N.K., Park K.Y. (2023). Skin aging from mechanisms to interventions: Focusing on dermal aging. Front. Physiol..

[107] Cui N., Hu M., Khalil R.A. (2017). Biochemical and Biological Attributes of Matrix Metalloproteinases. Prog. Mol. Biol. Transl. Sci..

[108] Favazzo L.J., Hendesi H., Villani D.A., Soniwala S., Dar Q.A., Schott E.M., Gill S.R., Zuscik M.J. (2020). The gut microbiome-joint connection: Implications in osteoarthritis. Curr. Opin. Rheumatol..

[109] Romero-Figueroa M.D.S., Ramírez-Durán N., Montiel-Jarquín A.J., Horta-Baas G. (2023). Gut-joint axis: Gut dysbiosis can contribute to the onset of rheumatoid arthritis via multiple pathways. Front. Cell. Infect. Microbiol..

[110] Takiishi T., Fenero C.I.M., Câmara N.O.S. (2017). Intestinal barrier and gut microbiota: Shaping our immune responses throughout life. Tissue Barriers.

[111] An J., Liu Y., Wang Y., Fan R., Hu X., Zhang F., Yang J., Chen J. (2022). The Role of Intestinal Mucosal Barrier in Autoimmune Disease: A Potential Target. Front. Immunol..

[112] Walrath T., Dyamenahalli K.U., Hulsebus H.J., McCullough R.L., Idrovo J.P., Boe D.M., McMahan R.H., Kovacs E.J. (2021). Age-related changes in intestinal immunity and the microbiome. J. Leukoc. Biol..

[113] Violi F., Cammisotto V., Bartimoccia S., Pignatelli P., Carnevale R., Nocella C. (2023). Gut-derived low-grade endotoxaemia, atherothrombosis and cardiovascular disease. Nat. Rev. Cardiol..

[114] Thevaranjan N., Puchta A., Schulz C., Naidoo A., Szamosi J.C., Verschoor C.P., Loukov D., Schenck L.P., Jury J., Foley K.P. (2017). Age-Associated Microbial Dysbiosis Promotes Intestinal Permeability, Systemic Inflammation, and Macrophage Dysfunction. Cell Host Microbe.

[115] Perler B.K., Chen B., Phelps E., Allegretti J.R., Fischer M., Ganapini V., Krajiceck E., Kumar V., Marcus J., Nativ L. (2020). Long-Term Efficacy and Safety of Fecal Microbiota Transplantation for Treatment of Recurrent Clostridioides difficile Infection. J. Clin. Gastroenterol..

[116] Hernandez C.J. (2017). The Microbiome and Bone and Joint Disease. Curr. Rheumatol. Rep..

[117] Kang D.Y., Sp N., Jo E.S., Rugamba A., Kim H.D., Kim I.H., Park J.C., Bae S.W., Jang K.J., Yang Y.M. (2021). Non-toxic sulfur inhibits LPS-induced inflammation by regulating TLR-4 and JAK2/STAT3 through IL-6 signaling. Mol. Med. Rep..

[118] Ahuja A., Kim E., Sung G.H., Cho J.Y. (2020). STAT3 Differentially Regulates TLR4-Mediated Inflammatory Responses in Early or Late Phases. Int. J. Mol. Sci..

[119] Kadomoto S., Izumi K., Mizokami A. (2021). Macrophage Polarity and Disease Control. Int. J. Mol. Sci..

[120] Sakai J., Cammarota E., Wright J.A., Cicuta P., Gottschalk R.A., Li N., Fraser I.D.C., Bryant C.E. (2017). Lipopolysaccharide-induced NF-κB nuclear translocation is primarily dependent on MyD88, but TNFα expression requires TRIF and MyD88. Sci. Rep..

[121] Ulivi V., Giannoni P., Gentili C., Cancedda R., Descalzi F. (2008). p38/NF-kB-dependent expression of COX-2 during differentiation and inflammatory response of chondrocytes. J. Cell. Biochem..

[122] Chung H.Y., Kim D.H., Lee E.K., Chung K.W., Chung S., Lee B., Seo A.Y., Chung J.H., Jung Y.S., Im E. (2019). Redefining Chronic Inflammation in Aging and Age-Related Diseases: Proposal of the Senoinflammation Concept. Aging Dis..

[123] Barber T.M., Kabisch S., Pfeiffer A.F.H., Weickert M.O. (2020). The Health Benefits of Dietary Fibre. Nutrients.

[124] Gentile C.L., Weir T.L. (2018). The gut microbiota at the intersection of diet and human health. Science.

[125] Krasnokutsky S., Oshinsky C., Attur M., Ma S., Zhou H., Zheng F., Chen M., Patel J., Samuels J., Pike V.C. (2017). Serum Urate Levels Predict Joint Space Narrowing in Non-Gout Patients with Medial Knee Osteoarthritis. Arthritis Rheumatol..

[126] Chelakkot C., Ghim J., Ryu S.H. (2018). Mechanisms regulating intestinal barrier integrity and its pathological implications. Exp. Mol. Med..

[127] Huang W.R., Tu J.X., Qiao A.Q., Chen L.J. (2022). GW842166X Alleviates Osteoarthritis by Repressing LPS-mediated Chondrocyte Catabolism in Mice. Curr. Med. Sci..

[128] Meng F., Lowell C.A. (1997). Lipopolysaccharide (LPS)-induced macrophage activation and signal transduction in the absence of Src-family kinases Hck, Fgr, and Lyn. J. Exp. Med..

[129] Page M.J., Kell D.B., Pretorius E. (2022). The Role of Lipopolysaccharide-Induced Cell Signalling in Chronic Inflammation. Chronic Stress.

[130] Huang Z., Kraus V.B. (2016). Does lipopolysaccharide-mediated inflammation have a role in OA?. Nat. Rev. Rheumatol..

[131] Fitzgerald K.A., Kagan J.C. (2020). Toll-like Receptors and the Control of Immunity. Cell.

[132] Yiu J.H., Dorweiler B., Woo C.W. (2017). Interaction between gut microbiota and toll-like receptor: From immunity to metabolism. J. Mol. Med..

[133] Lu J., Zhang H., Pan J., Hu Z., Liu L., Liu Y., Yu X., Bai X., Cai D., Zhang H. (2021). Fargesin ameliorates osteoarthritis via macrophage reprogramming by downregulating MAPK and NF-κB pathways. Arthritis Res. Ther..

[134] Bobacz K., Sunk I.G., Hofstaetter J.G., Amoyo L., Toma C.D., Akira S., Weichhart T., Saemann M., Smolen J.S. (2007). Toll-like receptors and chondrocytes: The lipopolysaccharide-induced decrease in cartilage matrix synthesis is dependent on the presence of toll-like receptor 4 and antagonized by bone morphogenetic protein 7. Arthritis Rheum..

[135] Ye X., Li H., Anjum K., Zhong X., Miao S., Zheng G., Liu W., Li L. (2022). Dual Role of Indoles Derived from Intestinal Microbiota on Human Health. Front. Immunol..

[136] Ruiz L., Delgado S., Ruas-Madiedo P., Sánchez B., Margolles A. (2017). Bifidobacteria and Their Molecular Communication with the Immune System. Front. Microbiol..

[137] Rivière A., Selak M., Lantin D., Leroy F., De Vuyst L. (2016). Bifidobacteria and Butyrate-Producing Colon Bacteria: Importance and Strategies for Their Stimulation in the Human Gut. Front. Microbiol..

[138] Lv X.C., Chen M., Huang Z.R., Guo W.L., Ai L.Z., Bai W.D., Yu X.D., Liu Y.L., Rao P.F., Ni L. (2021). Potential mechanisms underlying the ameliorative effect of Lactobacillus paracasei FZU103 on the lipid metabolism in hyperlipidemic mice fed a high-fat diet. Food Res. Int..

[139] Zhang S., Zhou J., Wu W., Zhu Y., Liu X. (2023). The Role of Bile Acids in Cardiovascular Diseases: From Mechanisms to Clinical Implications. Aging Dis..

[140] Liu J., Peng F., Cheng H., Zhang D., Zhang Y., Wang L., Tang F., Wang J., Wan Y., Wu J. (2023). Chronic cold environment regulates rheumatoid arthritis through modulation of gut microbiota-derived bile acids. Sci. Total Environ..

[141] Blevins H.M., Xu Y., Biby S., Zhang S. (2022). The NLRP3 Inflammasome Pathway: A Review of Mechanisms and Inhibitors for the Treatment of Inflammatory Diseases. Front. Aging Neurosci..

[142] Kelley N., Jeltema D., Duan Y., He Y. (2019). The NLRP3 Inflammasome: An Overview of Mechanisms of Activation and Regulation. Int. J. Mol. Sci..

[143] Perino A., Demagny H., Velazquez-Villegas L., Schoonjans K. (2021). Molecular Physiology of Bile Acid Signaling in Health, Disease, and Aging. Physiol. Rev..

[144] Zmora N., Suez J., Elinav E. (2019). You are what you eat: Diet, health and the gut microbiota. Nat. Rev. Gastroenterol. Hepatol..

[145] Myhrstad M.C.W., Tunsjø H., Charnock C., Telle-Hansen V.H. (2020). Dietary Fiber, Gut Microbiota, and Metabolic Regulation-Current Status in Human Randomized Trials. Nutrients.

[146] Dave M., Attur M., Palmer G., Al-Mussawir H.E., Kennish L., Patel J., Abramson S.B. (2008). The antioxidant resveratrol protects against chondrocyte apoptosis via effects on mitochondrial polarization and ATP production. Arthritis Rheum..

[147] Liu L., Gu H., Liu H., Jiao Y., Li K., Zhao Y., An L., Yang J. (2014). Protective effect of resveratrol against IL-1β-induced inflammatory response on human osteoarthritic chondrocytes partly via the TLR4/MyD88/NF-κB signaling pathway: An “in vitro study”. Int. J. Mol. Sci..

[148] Chen M.L., Yi L., Zhang Y., Zhou X., Ran L., Yang J., Zhu J.D., Zhang Q.Y., Mi M.T. (2016). Resveratrol Attenuates Trimethylamine-N-Oxide (TMAO)-Induced Atherosclerosis by Regulating TMAO Synthesis and Bile Acid Metabolism via Remodeling of the Gut Microbiota. mBio.

[149] Wang D., Zhang Z., Ju J., Wang X., Qiu W. (2011). Investigation of piceid metabolites in rat by liquid chromatography tandem mass spectrometry. J. Chromatogr. B.

[150] Yang H., Wang Y., Jin S., Pang Q., Shan A., Feng X. (2022). Dietary resveratrol alleviated lipopolysaccharide-induced ileitis through Nrf2 and NF-κB signalling pathways in ducks (*Anas platyrhynchos*). J. Anim. Physiol. Anim. Nutr..

[151] Zhuang Y., Wu H., Wang X., He J., He S., Yin Y. (2019). Resveratrol Attenuates Oxidative Stress-Induced Intestinal Barrier Injury through PI3K/Akt-Mediated Nrf2 Signaling Pathway. Oxidative Med. Cell. Longev..

[152] Omraninava M., Razi B., Aslani S., Imani D., Jamialahmadi T., Sahebkar A. (2021). Effect of resveratrol on inflammatory cytokines: A meta-analysis of randomized controlled trials. Eur. J. Pharmacol..

[153] Picca A., Faitg J., Auwerx J., Ferrucci L., D’Amico D. (2023). Mitophagy in human health, ageing and disease. Nat. Metab..

[154] Gu H., Li K., Li X., Yu X., Wang W., Ding L., Liu L. (2016). Oral Resveratrol Prevents Osteoarthritis Progression in C57BL/6J Mice Fed a High-Fat Diet. Nutrients.

[155] Jung Y.K., Park H.R., Cho H.J., Jang J.A., Lee E.J., Han M.S., Kim G.W., Han S. (2019). Degrading products of chondroitin sulfate can induce hypertrophy-like changes and MMP-13/ADAMTS5 production in chondrocytes. Sci. Rep..

[156] Zhang H., Qi L., Shen Q., Wang R., Guo Y., Zhang C., Richel A. (2022). Comparative Analysis of the Bioactive Compounds in Chicken Cartilage: Protective Effects of Chondroitin Sulfate and Type II Collagen Peptides Against Osteoarthritis Involve Gut Microbiota. Front. Nutr..

[157] Hu S., Wang J., Xu Y., Yang H., Wang J., Xue C., Yan X., Su L. (2019). Anti-inflammation effects of fucosylated chondroitin sulphate from Acaudina molpadioides by altering gut microbiota in obese mice. Food Funct..

[158] Bousbaine D., Fisch L.I., London M., Bhagchandani P., Rezende de Castro T.B., Mimee M., Olesen S., Reis B.S., VanInsberghe D., Bortolatto J. (2022). A conserved Bacteroidetes antigen induces anti-inflammatory intestinal T lymphocytes. Science.

[159] Tang R., Li L. (2021). Modulation of Short-Chain Fatty Acids as Potential Therapy Method for Type 2 Diabetes Mellitus. Can. J. Infect. Dis. Med. Microbiol..

[160] Belcaro G., Cesarone M.R., Errichi S., Zulli C., Errichi B.M., Vinciguerra G., Ledda A., Di Renzo A., Stuard S., Dugall M. (2008). Variations in C-reactive protein, plasma free radicals and fibrinogen values in patients with osteoarthritis treated with Pycnogenol. Redox Rep..

[161] Farid R., Rezaieyazdi Z., Mirfeizi Z., Hatef M.R., Mirheidari M., Mansouri H., Esmaelli H., Bentley G., Lu Y., Foo Y. (2010). Oral intake of purple passion fruit peel extract reduces pain and stiffness and improves physical function in adult patients with knee osteoarthritis. Nutr. Res..

[162] Dahiya D.K., Renuka, Puniya M., Shandilya U.K., Dhewa T., Kumar N., Kumar S., Puniya A.K., Shukla P. (2017). Gut Microbiota Modulation and Its Relationship with Obesity Using Prebiotic Fibers and Probiotics: A Review. Front. Microbiol..

[163] Lee S.H., Kwon J.Y., Jhun J., Jung K., Park S.H., Yang C.W., Cho Y., Kim S.J., Cho M.L. (2018). Lactobacillus acidophilus ameliorates pain and cartilage degradation in experimental osteoarthritis. Immunol. Lett..

[164] Yan R., Wang K., Wang Q., Jiang H., Lu Y., Chen X., Zhang H., Su X., Du Y., Chen L. (2022). Probiotic Lactobacillus casei Shirota prevents acute liver injury by reshaping the gut microbiota to alleviate excessive inflammation and metabolic disorders. Microb. Biotechnol..

[165] Lei M., Guo C., Wang D., Zhang C., Hua L. (2017). The effect of probiotic Lactobacillus casei Shirota on knee osteoarthritis: A randomised double-blind, placebo-controlled clinical trial. Benef. Microbes.

[166] Yu Z., Cao M., Peng J., Wu D., Li S., Wu C., Qing L., Zhang A., Wang W., Huang M. (2023). Lacticaseibacillus casei T1 attenuates Helicobacter pylori-induced inflammation and gut microbiota disorders in mice. BMC Microbiol..

[167] Collins K.H., Paul H.A., Reimer R.A., Seerattan R.A., Hart D.A., Herzog W. (2015). Relationship between inflammation, the gut microbiota, and metabolic osteoarthritis development: Studies in a rat model. Osteoarthr. Cartil..

[168] O-Sullivan I., Natarajan Anbazhagan A., Singh G., Ma K., Green S.J., Singhal M., Wang J., Kumar A., Dudeja P.K., Unterman T.G. (2022). Lactobacillus acidophilus Mitigates Osteoarthritis-Associated Pain, Cartilage Disintegration and Gut Microbiota Dysbiosis in an Experimental Murine OA Model. Biomedicines.

[169] Sarao L.K., Arora M. (2017). Probiotics, prebiotics, and microencapsulation: A review. Crit. Rev. Food Sci. Nutr..

[170] Henrotin Y., Patrier S., Pralus A., Roche M., Nivoliez A. (2021). Protective Actions of Oral Administration of Bifidobacterium longum CBi0703 in Spontaneous Osteoarthritis in Dunkin Hartley Guinea Pig Model. Cartilage.

[171] Chang S.L., Lin Y.Y., Liu S.C., Tsai Y.S., Lin S.W., Chen Y.L., Chen C.C., Ko C.Y., Chen H.T., Chen W.C. (2022). Oral Administration of Clostridium butyricum GKB7 Ameliorates Signs of Osteoarthritis in Rats. Cells.

[172] Schott E.M., Farnsworth C.W., Grier A., Lillis J.A., Soniwala S., Dadourian G.H., Bell R.D., Doolittle M.L., Villani D.A., Awad H. (2018). Targeting the gut microbiome to treat the osteoarthritis of obesity. JCI Insight.

[173] Zeng C.Y., Zhang Z.R., Tang Z.M., Hua F.Z. (2021). Benefits and Mechanisms of Exercise Training for Knee Osteoarthritis. Front. Physiol..

[174] Clauss M., Gérard P., Mosca A., Leclerc M. (2021). Interplay Between Exercise and Gut Microbiome in the Context of Human Health and Performance. Front. Nutr..

[175] Sohail M.U., Yassine H.M., Sohail A., Thani A.A.A. (2019). Impact of Physical Exercise on Gut Microbiome, Inflammation, and the Pathobiology of Metabolic Disorders. Rev. Diabet. Stud..

[176] Bo W., Zhou J., Wang K. (2018). Sodium butyrate abolishes the degradation of type II collagen in human chondrocytes. Biomed. Pharmacother..

[177] Bressa C., Bailén-Andrino M., Pérez-Santiago J., González-Soltero R., Pérez M., Montalvo-Lominchar M.G., Maté-Muñoz J.L., Domínguez R., Moreno D., Larrosa M. (2017). Differences in gut microbiota profile between women with active lifestyle and sedentary women. PLoS ONE.

[178] Zhou H., Li G., Wang Y., Jiang R., Li Y., Wang H., Wang F., Ma H., Cao L. (2021). Microbial Metabolite Sodium Butyrate Attenuates Cartilage Degradation by Restoring Impaired Autophagy and Autophagic Flux in Osteoarthritis Development. Front. Pharmacol..

[179] Hao X., Zhang J., Shang X., Sun K., Zhou J., Liu J., Chi R., Xu T. (2022). Exercise modifies the disease-relevant gut microbial shifts in post-traumatic osteoarthritis rats. Bone Jt. Res..

[180] Pasini E., Corsetti G., Assanelli D., Testa C., Romano C., Dioguardi F.S., Aquilani R. (2019). Effects of chronic exercise on gut microbiota and intestinal barrier in human with type 2 diabetes. Minerva Medica.

[181] Petriz B.A., Castro A.P., Almeida J.A., Gomes C.P., Fernandes G.R., Kruger R.H., Pereira R.W., Franco O.L. (2014). Exercise induction of gut microbiota modifications in obese, non-obese and hypertensive rats. BMC Genom..

[182] Lan H., Hong W., Qian D., Peng F., Li H., Liang C., Du M., Gu J., Mai J., Bai B. (2021). Quercetin modulates the gut microbiota as well as the metabolome in a rat model of osteoarthritis. Bioengineered.

[183] Feng K., Chen Z., Pengcheng L., Zhang S., Wang X. (2019). Quercetin attenuates oxidative stress-induced apoptosis via SIRT1/AMPK-mediated inhibition of ER stress in rat chondrocytes and prevents the progression of osteoarthritis in a rat model. J. Cell. Physiol..

[184] Yang L., Fan L., Wang K., Chen Y., Liang L., Qin X., Lu A., Cao P., Yu B., Guan D. (2021). Analysis of Molecular Mechanism of Erxian Decoction in Treating Osteoporosis Based on Formula Optimization Model. Oxidative Med. Cell. Longev..

[185] Jie L., Ma Z., Gao Y., Shi X., Yu L., Mao J., Wang P. (2023). The mechanism of palmatine-mediated intestinal flora and host metabolism intervention in OA-OP comorbidity rats. Front. Med..

[186] Chen Y., Lv J., Jia Y., Wang R., Zhang Z., Liu J., Jia C. (2020). Effect of Moxibustion on the Intestinal Flora of Rats with Knee Osteoarthritis Induced by Monosodium Iodoacetate. Evid.-Based Complement. Altern. Med..

[187] Jia Y.J., Li T.Y., Han P., Chen Y., Pan L.J., Jia C.S. (2022). Effects of different courses of moxibustion treatment on intestinal flora and inflammation of a rat model of knee osteoarthritis. J. Integr. Med..

[188] Zhang J., Li Q., Chang S. (2019). The effects of particle density in moxa smoke on the ultrastructure of knee cartilage and expressions of TNF-α, IL-1b, BAX, and Bcl-2 mRNA in a rat model for osteoarthritis. J. Cell. Biochem..

[189] Järvinen K., Vuolteenaho K., Nieminen R., Moilanen T., Knowles R.G., Moilanen E. (2008). Selective iNOS inhibitor 1400W enhances anti-catabolic IL-10 and reduces destructive MMP-10 in OA cartilage. Survey of the effects of 1400W on inflammatory mediators produced by OA cartilage as detected by protein antibody array. Clin. Exp. Rheumatol..

[190] Botha-Scheepers S., Watt I., Slagboom E., de Craen A.J., Meulenbelt I., Rosendaal F.R., Breedveld F.C., Huizinga T.W., Kloppenburg M. (2008). Innate production of tumour necrosis factor alpha and interleukin 10 is associated with radiological progression of knee osteoarthritis. Ann. Rheum. Dis..

[191] Gupta S., Allen-Vercoe E., Petrof E.O. (2016). Fecal microbiota transplantation: In perspective. Ther. Adv. Gastroenterol..

[192] Luckey D., Gomez A., Murray J., White B., Taneja V. (2013). Bugs & us: The role of the gut in autoimmunity. Indian J. Med. Res..

[193] Ianiro G., Bibbò S., Scaldaferri F., Gasbarrini A., Cammarota G. (2014). Fecal microbiota transplantation in inflammatory bowel disease: Beyond the excitement. Medicine.

[194] Pinn D.M., Aroniadis O.C., Brandt L.J. (2014). Is fecal microbiota transplantation the answer for irritable bowel syndrome? A single-center experience. Am. J. Gastroenterol..

[195] Vrieze A., Van Nood E., Holleman F., Salojärvi J., Kootte R.S., Bartelsman J.F., Dallinga-Thie G.M., Ackermans M.T., Serlie M.J., Oozeer R. (2012). Transfer of intestinal microbiota from lean donors increases insulin sensitivity in individuals with metabolic syndrome. Gastroenterology.

[196] Zeng J., Peng L., Zheng W., Huang F., Zhang N., Wu D., Yang Y. (2021). Fecal microbiota transplantation for rheumatoid arthritis: A case report. Clin. Case Rep..

[197] Huang Z., Chen J., Li B., Zeng B., Chou C.H., Zheng X., Xie J., Li H., Hao Y., Chen G. (2020). Faecal microbiota transplantation from metabolically compromised human donors accelerates osteoarthritis in mice. Ann. Rheum. Dis..

[198] So J.S., Song M.K., Kwon H.K., Lee C.G., Chae C.S., Sahoo A., Jash A., Lee S.H., Park Z.Y., Im S.H. (2011). Lactobacillus casei enhances type II collagen/glucosamine-mediated suppression of inflammatory responses in experimental osteoarthritis. Life Sci..

[199] Kwon J.Y., Lee S.H., Jhun J., Choi J., Jung K., Cho K.H., Kim S.J., Yang C.W., Park S.H., Cho M.L. (2018). The Combination of Probiotic Complex, Rosavin, and Zinc Improves Pain and Cartilage Destruction in an Osteoarthritis Rat Model. J. Med. Food.

[200] Wang S., Xu M., Wang W., Cao X., Piao M., Khan S., Yan F., Cao H., Wang B. (2016). Systematic Review: Adverse Events of Fecal Microbiota Transplantation. PLoS ONE.

[201] Novelle M.G., Naranjo-Martínez B., López-Cánovas J.L., Díaz-Ruiz A. (2025). Fecal microbiota transplantation, a tool to transfer healthy longevity. Ageing Res. Rev..

[202] DeFilipp Z., Bloom P.P., Torres Soto M., Mansour M.K., Sater M.R.A., Huntley M.H., Turbett S., Chung R.T., Chen Y.B., Hohmann E.L. (2019). Drug-Resistant *E. coli* Bacteremia Transmitted by Fecal Microbiota Transplant. N. Engl. J. Med..

